# Functional electrical stimulation to aid walking in patients with adrenomyeloneuropathy: A case study and observational series

**DOI:** 10.1002/jmd2.12254

**Published:** 2021-10-19

**Authors:** William Goodison, Fred Baron, Coralie Seary, Elaine Murphy, Robin Lachmann, Valerie L. Stevenson

**Affiliations:** ^1^ National Hospital for Neurology and Neurosurgery University College London Hospitals NHS Foundation Trust London UK; ^2^ Charles Dent Metabolic Unit National Hospital for Neurology and Neurosurgery London UK

**Keywords:** adrenomyeloneuropathy, foot drop, functional electrical stimulation, walking satisfaction, walking speed

## Abstract

Adrenomyeloneuropathy (AMN) is a rare inherited condition where affected individuals develop slowly progressive spastic paraparesis with a gradual decline in walking ability. There is no cure for AMN and treatment focuses on supportive measures and aids. One treatment option is functional electrical stimulation (FES), a treatment, approved by The National Institute for Health and Care Excellence (NICE), for managing foot drop in upper motor neuron disorders. Limited evidence exists for its use in AMN patients. We describe the effects of FES in an individual case and more broadly within a cohort of 21 patients successfully treated with FES. Patients with AMN referred for FES typically report frequent falls (71%) and foot drop (57%) as the most common barriers to walking. When using FES, walking speed at baseline (0.70 m/s [SD = 0.2]) was maintained at the 2‐year review (0.68 m/s [SD = 0.2]) with a persistent orthotic effect (improvement in walking speed when device on vs. off) seen from wearing FES over the same 2‐year period (11%–19%). Patient walking satisfaction (visual analogue scale: 0 – very dissatisfied; 10 – very satisfied) was also greater when comparing no‐FES versus FES over the same period (Year 1: 2.5 vs. 7.7; Year 2: 2.1 vs. 6.1). FES is not effective in all patients. Twelve patients referred found no benefit from the device; although there was no clear evidence, this was related to the degree of AMN associated peripheral neuropathy. However, FES is a safe, cost‐effective treatment option and should be considered, along with assessment in a multidisciplinary clinic, for all AMN patients with walking difficulties.

**Synopsis**

Functional electrical stimulation should be considered as a treatment option to maintain walking speed and walking satisfaction in patients with adrenomyeloneuropathy who present with regular falls, foot drop, deteriorating walking tolerance or walking confidence.

## 1 INTRODUCTION

X‐linked adrenoleukodystrophy (X‐ALD, OMIM 300100) is a rare genetic disorder, where a mutation in the *ABCD1* gene located on the X‐chromosome results in a disorder of peroxisomal fatty acid beta‐oxidation. It is one of the most frequently seen forms of adult leukodystrophy.^1^ The mutation leads to the build‐up of very long chain fatty acids (VLCFAs) in all tissues, but specifically the central nervous system and adrenal tissue.^2^ The pathophysiology of neurodegeneration and inflammation mediated by the accumulation of VLCFAs is not well understood but may relate to oxidative stress phenomena and energy depletion.^3^

There are a number of different phenotypes of X‐ALD. Cerebral ALD (cALD) is a progressive inflammatory demyelinating condition, which presents in about 35% of hemizygote males in childhood, between 3 and 10 years. It can also occur sporadically in adult males. It is normally associated with adrenal failure, which can precede the development of leukodystrophy. Adrenal failure can also present in boys and men without leukodystrophy. Adrenomyeloneuropathy (AMN) presents in adult males who did not succumb to cALD as children and is seen in nearly all patients with pathogenic *ABCD1* variants, most frequently presenting between the third and fourth decade.^4^ Female heterozygotes do not develop cALD and rates of adrenal failure are similar to the general population. They do, however, develop AMN, although it presents later in life and is more slowly progressive in women than in men.

Individuals with AMN develop a gradually progressive spastic paraparesis, in addition to bladder dysfunction, male erectile dysfunction, pain and sensory ataxia.^2^ AMN is a slowly progressive disorder, in contrast to cALD, caused by a non‐inflammatory axonopathy affecting both the spinal cord and peripheral nerves. The overall burden of peripheral neuropathy is likely underreported due to the predominance of central nervous system symptoms and signs due to spinal cord involvement. Before the advent of genetic sequencing, AMN was likely misdiagnosed as progressive multiple sclerosis or hereditary spastic paraparesis given the similarities in presentation and the sometimes unremarkable imaging. Some males with AMN will go on to develop cerebral involvement, adult onset cALD, which tends to have a slower and more faltering progression than childhood cALD.

There is no cure for AMN currently and treatment focuses on supportive measures aimed at mitigating the effects of the upper motor neuron injury, primarily spasticity and bladder management. Gait disorders are a common reason for presentation of AMN and walking speed has been shown to decline as the disease progresses.^5^ Given the rarity of AMN, trials looking at optimal support of walking in this patient group do not exist and people with AMN typically depend on walking aids including splints and orthoses to help manage the common feature of foot drop. At the National Hospital for Neurology and Neurosurgery (NHNN), we have a cohort of patients with AMN who have successfully been treated with functional electrical stimulation (FES) to help with their gait disorder and improve walking speed.

## 2 FUNCTIONAL ELECTRICAL STIMULATION

FES uses externally applied electrical stimulation to induce functional movement in weak muscles. It is a UK National Health Institute for Health and Clinical Excellence (NICE) approved treatment for the management of foot drop in upper motor neuron disorders and is most commonly used for correcting dropped foot in stroke and multiple sclerosis.^6^ Stimulation to the common peroneal nerve is timed to the swing phase of gait, causing dorsiflexion. FES has been shown to have positive effects on walking speed and stability as well as reducing the effort of walking and number of falls in patients.^7^, ^8^ Additional benefits from the use of FES include improvements in overactive bladder symptoms,^9^ as well as demonstrated improvement in quality of life in people with multiple sclerosis.^10^ Most research involving FES has focussed on patients with multiple sclerosis^11^ or stroke^12^ reflecting the increased prevalence of these conditions in adults with walking difficulties secondary to upper motor neuron lesions. However, FES has also been used successfully in a range of other neurological disorders, including hereditary spastic paraplegia,^13^ spinal cord injuries^14^ and more recently Parkinson's disease.^15^

At the NHNN, we use the Odstock Dropped Foot Stimulator (ODFS) Pace device (other devices are available, in the United States the FDA has approved the use of three FES machines including the one discussed here).^16^ This is a battery‐powered hip‐worn device, which connects via wires to surface electrodes on the patient's leg, and to a pressure‐sensitive footswitch in the patient's shoe. The footswitch synchronises the stimulation to the appropriate swing phase in the patient's gait. Electrode placement and several parameters of the stimulation are set up at initial assessment, depending on the patient's anatomy, impairments, comfort and features of their gait. Patients identify activity or participation goals for use of their FES, and they are monitored regularly in clinic. Dual channel devices can be used for people with bilateral foot drop, or for two muscle groups within one leg to manage foot drop with proximal weakness.

At each review, patients are timed over a 10 m distance both with and without the FES device to determine the ‘orthotic effect’ of the device (the difference in speed between off vs. on). The ‘total orthotic effect’ is the difference in speed between walking with the device and unassisted walking at baseline. In addition, FES has been shown to have a therapeutic effect on unassisted walking when the device has been worn for a period of time. This is known as the ‘training effect’ and may be secondary to muscle strengthening and motor re‐learning. This should be considered in the context of the neurological injury for which the patient is being treated. For example, in stroke both an orthotic and training effect has been reported,^17^ whereas in multiple sclerosis FES efficacy appears to be predominantly through an orthotic effect.^5^

## 3 CASE STUDY

A 40‐year‐old gentleman was referred to our FES walking clinic 10 years after his diagnosis with AMN. He initially presented with difficulty running, twitching legs and loss of balance. Of note there was a positive family history of ‘walking problems’ affecting his mother and brother, suggesting the possibility of X‐linked inheritance. The family were investigated and X‐ALD confirmed.

At our initial FES multidisciplinary review, he was taking baclofen 20 mg twice a day and desmopressin 20 mg in the evening. He lived with his wife and two children and there was no history of cognitive symptoms. In addition to his gait disturbance, he reported problems with his bladder, including frequency and urge incontinence. On review of his walking, he struggled with lifting his feet bilaterally and was hyperextending both knees. Due to the foot drop, he was tripping regularly and struggling to walk outdoors over uneven surfaces. His overall walking speed had reduced, although he felt that his walking distance was not impaired. He was mobilising with a single elbow crutch and Push Aequi splints for both ankles. Power throughout the lower limbs was 4 (MRC Muscle Strength Grading System 0–5) or greater in all muscle groups except for ankle dorsiflexion which was 2 bilaterally. Examination of tone (Ashworth scale) showed only slight spasticity (Grade 1) in the knee flexors and extensors bilaterally. On review of his gait, he had reduced dorsiflexion during swing phase bilaterally, causing him to scuff both feet. Knee flexion and hip flexion were also reduced during swing phase, more prominently on the left side giving him a stiff legged gait appearance.

His visual analogue scale (VAS) for walking satisfaction (0 – very dissatisfied; 10 – extremely satisfied) was 1 at baseline. His baseline walking speed was assessed with a 10 m timed walk (10mTW) and recorded as 1.02 m/s. He was set up with a dual channel stimulator targeting bilateral foot drop. Stimulation resulted in improved foot clearance during swing phase. His walking speed increased immediately to 1.10 m/s, an 8% improvement. He trialled the device for 6 weeks and at this point he reported increased confidence with his walking, with fewer trips and falls. He initially reported increased spasm triggered by stimulation, but these settled with FES use. His VAS for walking satisfaction increased to 8 (0 – very dissatisfied; 10 – extremely satisfied) whilst wearing his FES. His 10mTW off FES at this point was 1.10 m/s and with the device his speed had increased to 1.24 m/s (a 22% increase in speed from his initial baseline pre‐FES). The improvement in his walking speed without the device suggested a training effect of using the FES daily. He set new goals for his rehabilitation, which included: (1) to walk at the same speed as his family; (2) to play basic football with his son.

This gentleman continued with FES use and despite the progressive nature of AMN at his 2‐year review he demonstrated an 18% increase in his speed of walking on his 10mTW when comparison was made between FES off and on (0.88 m/s vs. 1.04 m/s). However, his best speed whilst wearing FES was now only 2% greater than his original baseline and his walking speed without FES had reduced from 1.02 m/s at baseline to 0.88 m/s over that period (−14% change) demonstrating disease progression. His VAS for walking satisfaction with FES was now 6 (0 – very dissatisfied; 10 – extremely satisfied). Although the device continued to provide good foot clearance for him, he felt increasingly fatigued and reliant on the FES to maintain his walking. He continued to use his splints bilaterally to help with ankle stability.

**FIGURE 1:**
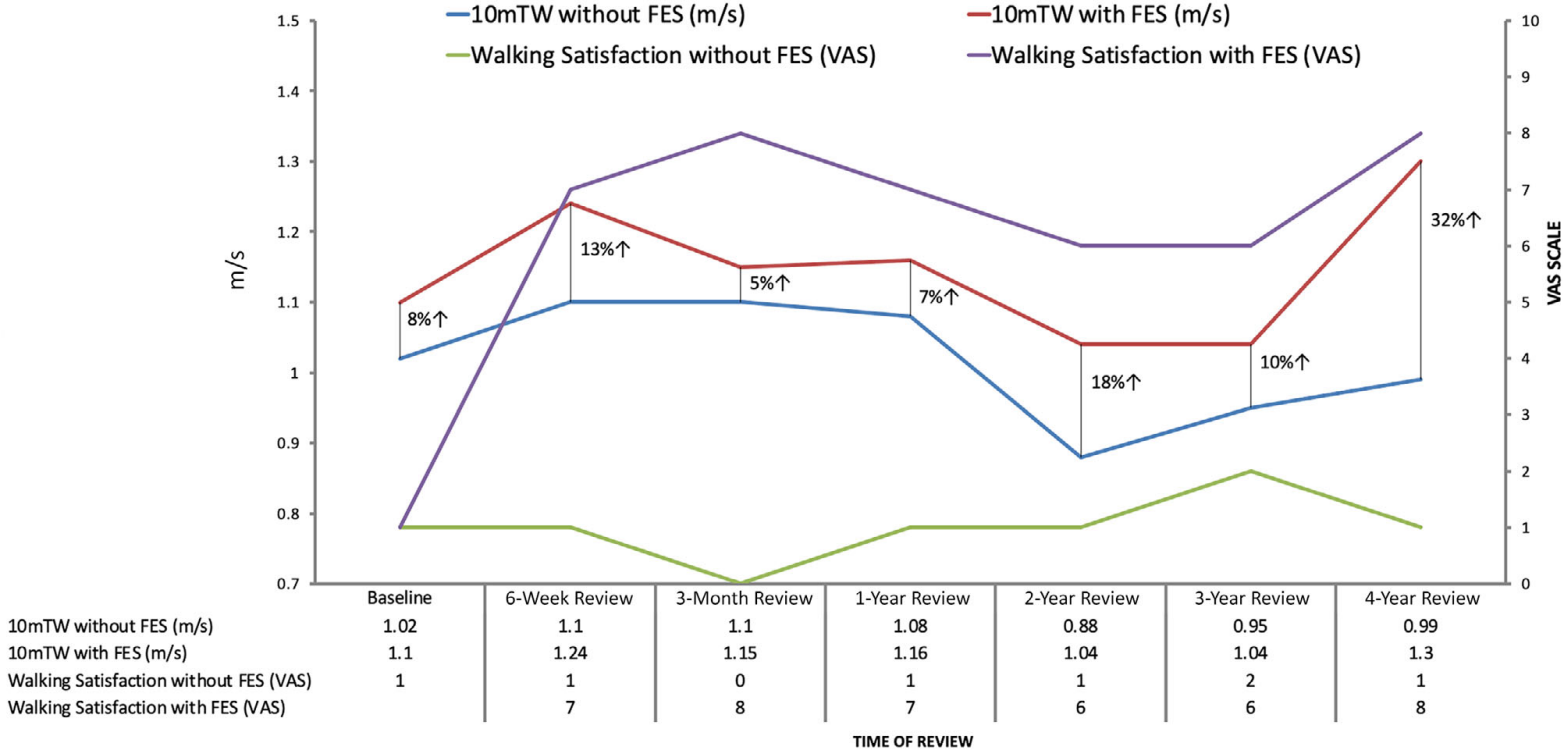
Case report: 10 m timed walk (10mTW) and walking satisfaction (VAS) with and without FES over a 4‐year period in our patient. Despite a gradual decline in walking speed, use of FES continued to demonstrate a good orthotic effect (% improvement) at each assessment. The patient's satisfaction with their waking on FES was also consistently greater despite the progressive nature of the disease. FES, functional electrical stimulation

He has continued to use the FES and has now been wearing the device for over 5 years. Over that time, his VAS scores for walking with FES have remained stable (6–8). His baseline walking speed over time has been on a downward trend, in line with his disease progression. However, he continues to get a good orthotic effect from the FES (Figure 1).

## 4 NHNN AMN COHORT

**FIGURE 2:**
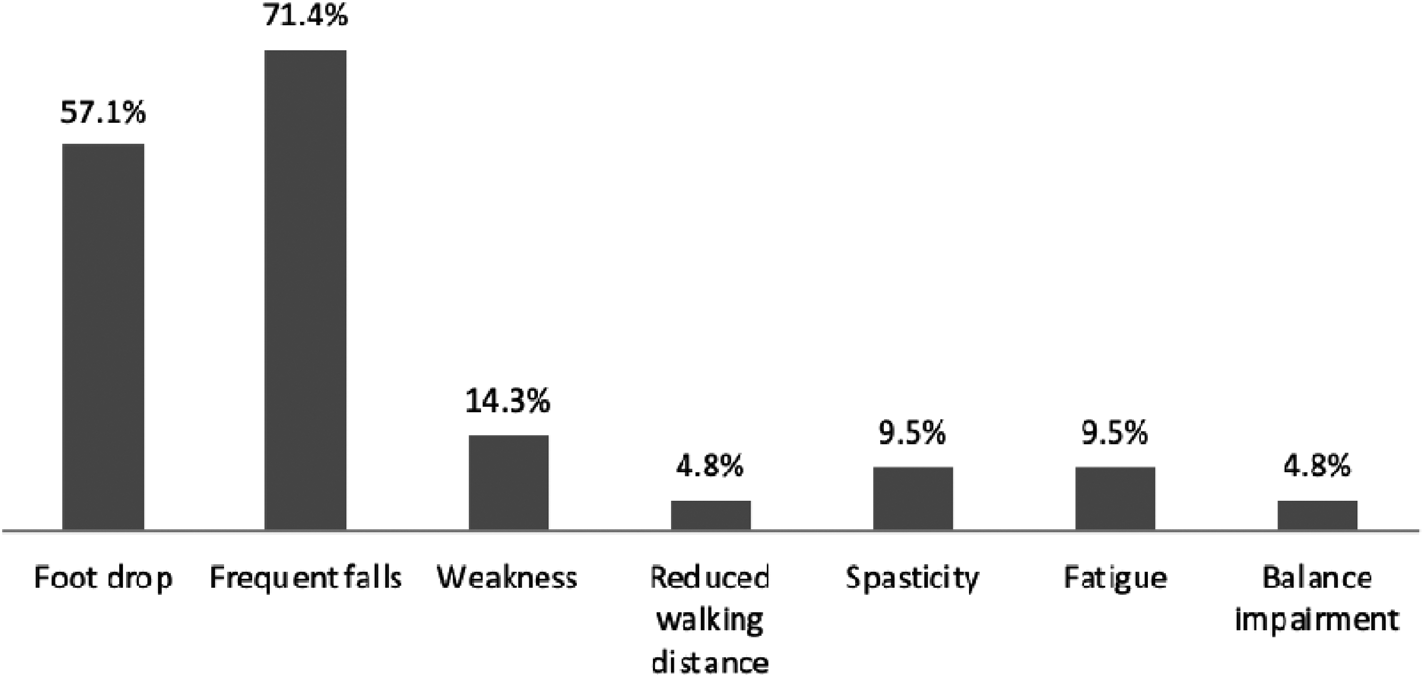
Patient reported issues at baseline assessment

Between 2011 and 2021, 33 patients with AMN have been referred to our FES service for assessment. Twenty‐one (64%) patients progressed to treatment and 12 (36%) did not. Baseline characteristics for patients are presented in Table 1. The most common patient reported issues with walking were frequent falls (71%) and foot drop (57%; unilateral or bilateral) (Figure 2). Data from nerve conduction studies (NCSs) were available for 17 patients from the total cohort (11 patients in the responder group; 6 patients in the non‐responder group) as well as clinical records for evidence of sensory impairments to vibration or proprioception (Table 1). Similar levels of abnormalities in clinical evidence of a loss of distal vibration or proprioception or in formal NCSs were seen in both groups.

**TABLE 1:** Baseline demographics of AMN patients referred for FES assessment

	Total cohort (n = 33)	Responders (n = 21)	Non‐responders (n = 12)
Male	21 (64%)	15 (71%)	6 (50%)
Female	12 (36%)	6 (29%)	6 (50%)
Age at presentation, years (SD)	48.4 (10.0)	47.2 (10.8)	50.5 (8.5)
Male	43.7 (8.7)	43.2 (9.6)	45.0 (6.6)
Female	56.6 (6.1)	57.2 (6.3)	56.0 (6.4)
Age at diagnosis, years (SD)	40.8 (13.2)	38.9 (7.9)	45.2 (9.3)
Male	33.2 (6.3)	30.7 (5.1)	40.5 (80.4)
Female	46.8 (9.3)	44.5 (9.9)	49.1 (9.0)
Walking aid use			
None (%)	12 (36%)	6 (29%)	6 (50%)
Single stick/crutch (%)	15 (46%)	11 (52%)	4 (33%)
Dual aids/walking frame (%)	6 (18%)	4 (19%)	2 (17%)
Single channel stimulator	N/A	13 (62%)	N/A
Dual channel stimulator	N/A	8 (38%)	N/A
NCSs – Evidence of large fibre neuropathy	(n = 17)	(n = 11)	(n = 6)
Yes	8 (47%)	5 (45%)	3 (50%)
No	9 (53%)	6 (55%)	3 (50%)
Clinical evidence of peripheral neuropathy			
Yes	16 (94%)	10 (91%)	6 (100%)
No	1 (6%)	1 (9%)	0

Abbreviations: AMN, adrenomyeloneuropathy; FES, functional electrical stimulation; NCSs, nerve conduction studies.

Of the 21 patients who responded to treatment with FES, 13 were set up with a single channel stimulator at first visit (62%) and 8 with dual stimulators (38%). Two patients using single channel stimulators were switched to dual channel at 3 months, and one patient was switched from dual to single channel at 3 months due to difficulty using the device. Baseline mean walking speed (10mTW) was 0.7 m/s (SD = 0.2). The mean increase in walking speed at initial set up was 0.08 m/s, an 11% increase. Baseline average VAS for walking satisfaction at baseline was 2 (0 – very dissatisfied; 10 – extremely satisfied) (Table 2). Data on walking speed and VAS score were missing for one patient at baseline.

**TABLE 2:** Walking satisfaction and speed over 10 m walk (10mTW)

	Baseline	1‐year review	2‐year review
	(n = 20)	(n = 9)	(n = 7)
Walking satisfaction, VAS (SD)			
Without FES	2 (1.5)	2.5 (2.1)	2.1 (2.3)
With FES	N/A	7.7 (1.0)	6.1 (1.8)
Mean walking speed, m/s (SD)			
Without FES	0.7 (0.2)	0.63 (0.3)	0.57 (0.2)
With FES	0.78 (0.2)	0.71 (0.3)	0.68 (0.2)
% Improvement	11%	13%	19%

Abbreviations: FES, functional electrical stimulation; VAS, visual analogue scale.

Twelve patients (36%) did not progress to treatment with FES. Six (50%) had no effect from the FES despite high currents and four of these also reported pain at the higher currents needed to try and stimulate the nerve. In three (25%) patients, FES was not indicated because walking function was only minimally impaired. Two (17%) declined treatment with FES despite a positive effect, in both cases the patients were only mobilising short distances and felt the FES did not add significant benefit to their functional goals. One (8%) patient did not attend their appointment and declined further assessment. Data on walking speed for the group who did not progress with FES were not collected. Six (50%) were offered alternative devices to help with walking (insoles/ankle splints/ankle foot orthoses). One patient was advised to reduce their baclofen medication due to lower limb weakness affecting their walking.

### 4.1 Length of use

Six patients (29%) discontinued FES after a 3‐month trial. The most common reasons given for stopping were (multiple answers given): ‘no benefit’ (67%); ‘too effortful to set up’ (67%); ‘painful’ (17%); ‘worsening spasms’ (17%). Two (10%) patients were transferred to local services at their 3‐month follow‐up to continue FES treatment and were lost to follow up.

At 1 year a further two patients stopped treatment with FES both reporting that FES was ‘too effortful to set up’ with inconvenience outweighing any benefit. One of these patients also struggled as she found it cosmetically unacceptable to wear FES with a skirt because of the presence of the external wires.

Eleven patients completed at least 2 years of treatment at NHNN, although data collection was incomplete in four of these patients. When wearing FES, the VAS scores for Walking Satisfaction at ‘1‐Year Review’ was 7.7, and 6.1 at ‘2‐Year Review’ (Table 2). At 1 year, the mean percentage increase for walking speed with and without FES use was 13% and at 2 years was 19% (Table 2). There were no clinically adverse events reported to using FES amongst this cohort.

## 5 DISCUSSION

AMN is a progressive disorder, with no current disease modifying treatments available. Progressive neurological diseases have been shown to have a negative impact on health‐related quality of life^18^; therefore, targeted symptomatic treatments are essential for the long‐term management of these patients. FES has been shown to be a cost effective treatment of foot drop, which improves quality of life in patients with upper motor neuron lesions.^19^ However, walking speed alone does not correlate with improvements in patient reported quality of life,^10^ and should not be the sole determining factor on decisions about treatment; walking speed is simply one element of walking. The VAS scale,^20^ which considers walking satisfaction, encompasses the patient's subjective experience of their walking which may include speed, smoothness of gait and confidence for example. As our case study showed, functional goals, such as returning to playing football with family, are much more relevant to patients and are likely to impact significantly on a patient's mental well‐being as they deal with a progressive disorder.

Our cohort of patients continued to demonstrate a good ‘orthotic effect’ from FES even at their 2‐year review. These results are in keeping with studies looking at other progressive neurological disorders such as multiple sclerosis and hereditary spastic paraparesis which similarly did not find any evidence of a ‘training effect’ from the FES,^5^ compared with single insult disorders such as stroke. Overall walking speeds fell over the 2‐year period as would be expected in a progressive disorder such as AMN. This is in line with Huffnagel and colleagues^7^ who demonstrated declining walking function over a 2‐year follow‐up period in male patients with AMN. In this study, the ‘timed up‐and‐go’ (seconds) and 6‐min walk test (metres) were used to assess function and reduced by 0.82 s (*p* value = 0.032) and 19.67 m (*p* value = 0.019), respectively. In our cohort, we also observed a drop in walking speed over 2 years. However, FES appeared to mitigate this loss over the same time period, despite the progressive nature of the disease, and maintained walking speed close to the patient's baseline level. This drop in speed is also accompanied by a small drop in the VAS scores in Year 2 compared to Year 1 which would also be in line with on‐going disease progression.

For this cohort of patients with AMN, continued walking with FES is also likely to have significant positive effects on maintaining lower limb strength and spasticity management similar to other patient groups,^21^ this has implications for the medical management of these patients by avoiding the sedating side effects of systemic anti‐spasmodic treatments. FES has also been shown to improve urinary incontinence in patients with progressive neurological conditions, such as multiple sclerosis.^9^ Whilst not included in this review, further research looking at this in patients with AMN could yield similar results and deserves attention, given the similarities observed in walking effect when using FES.

FES can be difficult initially for patients to set up and tolerate, as shown by the numbers stopping it after only 3 months. ‘Difficulty using equipment’, ‘not effective’ and ‘painful’ have frequently been quoted in studies that have captured reasons for stopping FES.^19^ Just over one third of patients referred with AMN did not respond to, or were inappropriate for, FES which is line with our normal referral data to the service (audit data 70% rate of setup of FES from assessment; V. L. Stevenson, personal communication). This is interesting as it is important to recognise that unlike other commonly treated neurological disorders, AMN has a combination of both upper and lower motor neuron features. As FES depends on a functional peripheral nervous system to relay stimulation to muscles, it would therefore not be surprising if patients with greater peripheral involvement stimulation had a suboptimal response or required a higher current resulting in discomfort or exacerbation of sensory symptoms. However, this was not seen in our series and should certainly not discourage the consideration of FES for this patient group. Only 17 (52%) of the patients identified in this report had historical NCSs and the incidence of large fibre peripheral neuropathy was similar for both groups although the data were variable and not consistent with regards to the timing of the investigations and initiation of FES. Chaudhry et al.^22^ found 53% of patients with AMN (n = 99) had evidence of abnormal peroneal nerve conduction velocity which is consistent with our data presented here.

Understanding when to refer for FES is important for clinicians managing patients with AMN. Whilst not the primary outcome of our data collection, differences between the two cohorts of patients referred can be drawn. In general, the group responding to FES appeared to have a younger age at diagnosis, with the difference particular marked between the male responders and non‐responders. This could be explained by the fact that a diagnosis of AMN later in life is more likely to represent a slower progression of disease with milder gait abnormalities and thus the benefits of FES are not as obvious in these patients. This area would warrant further investigation to gain an understanding of whether patients who initially declined the use of FES returned to it later in life, but unfortunately such analysis is not possible with the data presented here. Additionally, of patients responding to FES, 71% were using at least one walking aid at presentation, compared to 50% in the non‐responders. This may suggest less severe disease within the non‐responder cohort at time of referral. Understanding how walking aid use changes over time would also be of interest to clinicians managing patients with AMN and should be considered in future studies in this group.

Our cohort did not receive any additional physiotherapy input beyond advice and review delivered in the physiotherapy led follow‐up FES clinics. Previous work has suggested improved efficacy of FES when combined with physiotherapy,^23^ although this was specifically in patients with stroke rather than progressive neurological disorders. It would warrant further study in the future to quantify whether the addition of intensive therapy can provide a clear and sustained training effect in AMN patients.

Whilst none of our cohort of male patients with AMN demonstrated cognitive impairment (cALD) at their first assessment or during the study period, it is recognised that in a small percentage of patients with AMN this will develop and this in turn may have implications for setting up of the FES device and should be considered as part of any decision to trial FES in this group.

## 6 CONCLUSIONS

The data presented here is obviously limited by the small numbers included. Data collection was prospective, but not complete for several patients in our cohort. It is therefore impossible to draw any significant findings on efficacy although comparisons with data from Huffnagel et al.^5^ suggest a benefit to using FES for maintaining walking function for a longer period of time and patients reported increased satisfaction with walking. AMN by its nature is progressive and so the effectiveness of FES in maintaining walking will decrease over time due to central and peripheral effects of the disease. Although peripheral involvement should be considered when assessing the effectiveness of FES in this group of patients both at trial and over time, it should not preclude referral and assessment. There is no evidence from this observational study that NCSs are helpful in predicting the usefulness of FES in improving walking function.

Given the cost effectiveness and benefits of treatment demonstrated in other progressive neurological disorders, we feel that FES should be considered for both male and female patients with AMN in the long‐term management of their walking impairments. All AMN patients with walking difficulty would benefit from a review in a multidisciplinary clinic to consider treatments such as FES in addition to other walking adjuncts when FES is not effective.

### 6.1 Key points

Adrenomyeloneuropathy (AMN) is a slowly progressive disorder caused by a non‐inflammatory axonopathy affecting both the spinal cord and peripheral nerves.

Progressive difficulty with walking and falls is a common feature of AMN.

Functional electrical stimulation (FES) is a UK National Health Institute for Health and Clinical Excellence (NICE) approved treatment for the management of foot drop in upper motor neuron disorders. Externally applied electrical stimulation to the common peroneal nerve is timed to the swing phase of gait, causing dorsiflexion.

Patients with AMN reporting tripping, falling, reduced walking tolerance or confidence will benefit from multidisciplinary assessment for FES.

## CONFLICT OF INTEREST

William Goodison, Fred Baron, Coralie Seary, Elaine Murphy and Robin Lachmann declare that they have no conflicts of interest. Valerie L. Stevenson reports personal fees from Medtronic Advisory Board, personal fees from GW Pharmaceuticals Advisory Board and personal fees from European Continuing medical Training, outside the submitted work.

## ETHICAL APPROVAL

No ethics approval was required for this review as data were collected as part of routine appointments within the FES service and analysed retrospectively. All procedures followed were in accordance with the ethical standards of the responsible committee on human experimentation (University College Hospital London and UK) and with the Helsinki Declaration of 1975, as revised in 2000.

## PATIENT CONSENT STATEMENT

Informed consent was obtained from all patients for whom identifying information is included in this article.

## Data Availability

The datasets generated during and/or analysed during the current study are available from the corresponding author on reasonable request.
